# Supplementary material for: A mechanistic basis for CD8+ T cell expansion sensitivity as a predictor of HIV post-treatment control
Source: bioRxiv. 2026 Sep 1:2026.08.28.747758. Preprint. [Version 1] doi: 10.64898/2026.08.28.747758 (PMC13684515; doi:10.64898/2026.08.28.747758)
Supplement: Supplementary file 1 [file NIHPP2026.08.28.747758v1-supplement-1.pdf]

bioRxiv preprint doi: https://doi.org/10.64898/2026.08.28.747758; this version posted September 1, 2026. The copyright holder for this preprint (which was not certified by peer review) is the author/funder. This article is a US Government work. It is not subject to copyright under 17 USC 105 and is also made available for use under a CC0 license.

Supplementary Materials

S1 Text. Comparison of several variations of the Conway-Perelson model.

To add a non-cytolytic effect to the main model, we make the modification

$$\frac{dV}{dt} = \frac{pI}{1 + m_2 E} - cV.$$

Here, m_2 represents the magnitude of the effect of effector cells on reducing the rate of viral production. The model fit comparison in Table S1 shows that the main model without a noncytolytic effect is the preferred model.

Model	-2LL	BICc
Conway – Perelson model	78.02	180.93
Main model	**73.16**	**159.55**
Main model with non-cytolytic effector function	80.15	174.81

Table S1. Model comparison.

S2 Text. Best fit parameters for the main model.

The best fit population and individual parameters are given in Tables S2 and S3, respectively.

Parameter	Population estimate	95% CI	Individual estimate (min – max)
L_0 (cells/mL)	10	NaN	[10, 10]
$-\log_{10}\beta$ *(mL viral copies⁻¹ day⁻¹)*	8.5	[8.42, 8.58]	[8.50, 8.50]
λ_E *(cells mL^{-1} day^{-1})*	1.34	[0.76, 2.25]	[0.52, 2.04]
$\log_{10} K_B$ (NC)	3.77	[2.98, 4.55]	[3.65, 3.75]
$\log_{10} K_B$ (PIC)	2.46	[1.99, 2.92]	[1.77, 3.25]
d_E (day^{-1})	0.049	[0.028, 0.086]	[0.018, 0.084]
m *(mL cell^{-1} day^{-1})*	0.023	NaN	[0.022, 0.025]
$\log_{10} p$ (day^{-1})	4.12	[4.04, 4.2]	[4.10, 4.14]
t_a (days)	87.54	[59.38, 129.04]	[28.23, 170.36]
f_E	0.044	NaN	[0.039, 0.050]

Table S2. Best fit (population) parameters for the main model. Individual parameters are presented in Table S3. NaN indicates Monolix could not estimate the value.

bioRxiv preprint doi: https://doi.org/10.64898/2026.08.28.747758; this version posted September 1, 2026. The copyright holder for this preprint (which was not certified by peer review) is the author/funder. This article is a US Government work. It is not subject to copyright under 17 USC 105 and is also made available for use under a CC0 license.

PID	L_0	$-log_{10}\,\beta$	λ_E	$log_{10}\,K_B$	d_E	m	$log_{10}\,p$	t_a	f_E
10227	10	8.50	1.23	1.77	0.045	0.022	4.12	136.89	0.041
71190	10	8.50	0.93	2.15	0.042	0.024	4.12	43.71	0.047
47019	10	8.50	1.23	2.28	0.047	0.025	4.11	96.80	0.050
72210	10	8.50	1.71	2.35	0.084	0.024	4.1	160.18	0.047
48758	10	8.50	0.52	2.87	0.018	0.023	4.12	170.36	0.046
30988	10	8.50	1.66	3.25	0.054	0.024	4.11	69.32	0.045
42894	10	8.50	1.52	3.65	0.043	0.022	4.14	141.16	0.039
31092	10	8.50	1.78	3.67	0.061	0.023	4.13	28.23	0.043
35933	10	8.50	2.04	3.75	0.084	0.023	4.14	74.43	0.041

Table S3. Best fit (individual) parameters for the main model. Population parameters and parameter units are presented in Table S2.

S3 Text. Robustness of correlations across perturbations to the model fitting procedure.

During the model testing phase, we examined various perturbations to the model fitting. While different assumptions affect the goodness of fit (and/or BICc score), the correlation between $1/K_B$ and markers of immune stemness at the time of rebound is robust (Tables S4-5).

**Model**	**-2LL**	**BICc**	**(r) 1/K_B : Ki67**	R^2	***p*-value**	**(r) 1/K_B : TCF-1**	R^2	***p*-value**	
Main Model (L_0)	57.80	144.2	0.80	0.64	0.017	0.84	0.71	0.008	
Main Model (β)	70.06	156.5	0.81	0.66	0.014	0.80	0.64	0.017	
Main Model (λ_E)	56.43	142.8	0.80	0.64	0.017	0.85	0.72	0.008	
Main Model (d_E)	55.84	142.2	0.78	0.60	0.024	0.74	0.55	0.036	
Main Model (m)	67.27	153.7	0.80	0.65	0.016	0.75	0.57	0.031	
Main Model (p)	70.19	156.6	0.80	0.64	0.018	0.75	0.57	0.031	
Main Model (t_a)	158.6	245.0	0.84	0.71	0.009	0.67	0.44	0.071	
Main Model (f_E)	66.14	152.5	0.77	0.59	0.026	0.74	0.55	0.036	
Main Model ($\lambda_E\ L_0$)	57.49	141.7	0.80	0.64	0.017	0.84	0.71	0.008	
Main Model ($\lambda_E\ \beta$)	55.45	139.7	0.77	0.60	0.024	0.74	0.55	0.036	
Main Model ($\lambda_E\ d_E$)	51.32	135.5	0.76	0.57	0.030	0.72	0.51	0.046	
Main Model ($\lambda_E\ m$)	76.95	161.2	0.84	0.71	0.009	0.65	0.42	0.084	
Main Model ($\lambda_E\ p$)	53.94	138.1	0.77	0.59	0.026	0.73	0.54	0.038	
Main Model ($\lambda_E\ t_a$)	134.3	218.4	0.78	0.60	0.023	0.75	0.57	0.031	
Main Model ($\lambda_E\ f_E$)	66.14	150.6	0.78	0.61	0.022	0.86	0.73	0.007	
			0.79	0.63	0.019	0.76	0.59	0.033	Mean
			0.78	0.60	0.017	0.74	0.55	0.013	Q_1
			0.80	0.65	0.024	0.82	0.68	0.037	Q_3
			0.80	0.64	0.018	0.75	0.57	0.031	Median

Table S4. Main model fitting with varying the number of fitting parameters. (r) is Pearson's correlation coefficient. Main model refers to the main text model with a covariate on K_B. The parameters in the parentheses are fitted without random effects. First, we tested removing the

bioRxiv preprint doi: https://doi.org/10.64898/2026.08.28.747758; this version posted September 1, 2026. The copyright holder for this preprint (which was not certified by peer review) is the author/funder. This article is a US Government work. It is not subject to copyright under 17 USC 105 and is also made available for use under a CC0 license.

random effects on each parameter. We selected the best variation, based on -2LL and the correlation strength. Then, we repeat the process by removing the random effects for a second parameter from the best variation. After two rounds (two parameters without random effects), removing the random effects from a third parameter does not improve the BICc by more than 5 points.

Fixed parameter	(r) $1/K_B$: Ki67	R^2	p-value	(r) $1/K_B$: TCF-1	R^2	p-value	
$a_L \times 2$	0.79	0.62	0.020	0.73	0.54	0.039	
$a_L \times 5$	0.83	0.68	0.012	0.83	0.69	0.011	
$a_L \div 2$	0.77	0.59	0.026	0.74	0.54	0.037	
$a_L \div 5$	0.76	0.58	0.029	0.74	0.55	0.034	
$b \times 2$	0.81	0.65	0.016	0.84	0.70	0.009	
$b \times 5$	0.82	0.67	0.013	0.77	0.59	0.025	
$b \div 2$	0.77	0.60	0.025	0.74	0.55	0.036	
$b \div 5$	0.77	0.60	0.025	0.74	0.55	0.036	
$c \times 2$	0.78	0.61	0.023	0.74	0.54	0.037	
$c \times 5$	0.80	0.64	0.018	0.85	0.73	0.007	
$c \div 2$	0.83	0.68	0.012	0.61	0.37	0.109	
$c \div 5$	0.80	0.65	0.016	0.52	0.27	0.191	
$d_L \times 2$	0.76	0.58	0.028	0.74	0.55	0.036	
$d_L \times 5$	0.76	0.58	0.028	0.74	0.55	0.035	
$d_L \div 2$	0.80	0.64	0.017	0.84	0.71	0.009	
$d_L \div 5$	0.80	0.64	0.018	0.73	0.53	0.039	
$d_T \times 2$	0.80	0.63	0.018	0.85	0.72	0.007	
$d_T \times 5$	0.81	0.65	0.016	0.85	0.72	0.008	
$d_T \div 2$	0.75	0.56	0.032	0.49	0.24	0.222	
$d_T \div 5$	0.72	0.51	0.045	0.68	0.46	0.066	
$\delta \times 2$	0.80	0.64	0.017	0.85	0.71	0.008	
$\delta \times 5$	0.85	0.72	0.008	0.54	0.29	0.166	
$\delta \div 2$	0.77	0.60	0.024	0.74	0.55	0.035	
$\delta \div 5$	0.76	0.58	0.027	0.74	0.55	0.035	
$f_L \times 2$	0.82	0.68	0.012	0.77	0.60	0.025	
$f_L \times 5$	0.76	0.58	0.029	0.74	0.55	0.036	
$f_L \div 2$	0.82	0.67	0.013	0.77	0.59	0.025	
$f_L \div 5$	0.82	0.68	0.012	0.77	0.59	0.027	
$\lambda_T \times 2$	0.83	0.68	0.012	0.61	0.37	0.107	
$\lambda_T \times 5$	0.73	0.54	0.039	0.67	0.44	0.071	
$\lambda_T \div 2$	0.80	0.65	0.016	0.85	0.72	0.008	
$\lambda_T \div 5$	0.81	0.65	0.016	0.84	0.71	0.009	
$p \times 2$	0.81	0.65	0.015	0.80	0.65	0.016	
$p \times 5$	0.86	0.73	0.007	0.76	0.58	0.028	
$p \div 2$	0.82	0.67	0.013	0.78	0.60	0.024	
$p \div 5$	0.76	0.57	0.030	0.74	0.55	0.036	
	0.79	0.63	0.02	0.74	0.56	0.046	Mean
	0.77	0.59	0.01	0.74	0.54	0.01	Q_1
	0.82	0.67	0.03	0.81	0.66	0.04	Q_3
	0.80	0.64	0.02	0.74	0.55	0.04	Median

Table S5. Main model fitting with fixed parameters varied by factors of 2 and 5. (r) is Pearson's correlation coefficient. Main model refers to the main text model with a covariate on K_B.

bioRxiv preprint doi: https://doi.org/10.64898/2026.08.28.747758; this version posted September 1, 2026. The copyright holder for this preprint (which was not certified by peer review) is the author/funder. This article is a US Government work. It is not subject to copyright under 17 USC 105 and is also made available for use under a CC0 license.

S4 Text. Viral set point correlation to stemness marker.

Previously, we established that for the main model there is a linear relationship between K_B and the viral set point [38], shown in Equation S1, and demonstrated in Fig. S1. We further observe that due to the small variations between the parameters other than K_B across participants (Fig. 3a), the term in the parentheses of Equation S1 is approximately constant among participants. This gives the last approximation in equation S1, which is the basis of the correlation between K_B and the viral set point.

$$V_{\text{set point}} \approx \left(\frac{p}{c} \frac{m\lambda_E - \left((1-f_L)\beta \left(\frac{p}{c}\right)\left(\frac{\lambda_T}{d_T}\right) - \delta \right) d_E}{\left((1-f_L)\beta \left(\frac{p}{c}\right)\left(\frac{\lambda_T}{d_T}\right) - \delta \right)(d_E - b) - m\lambda_E} \right) K_B \approx \alpha K_B \tag{S1}$$

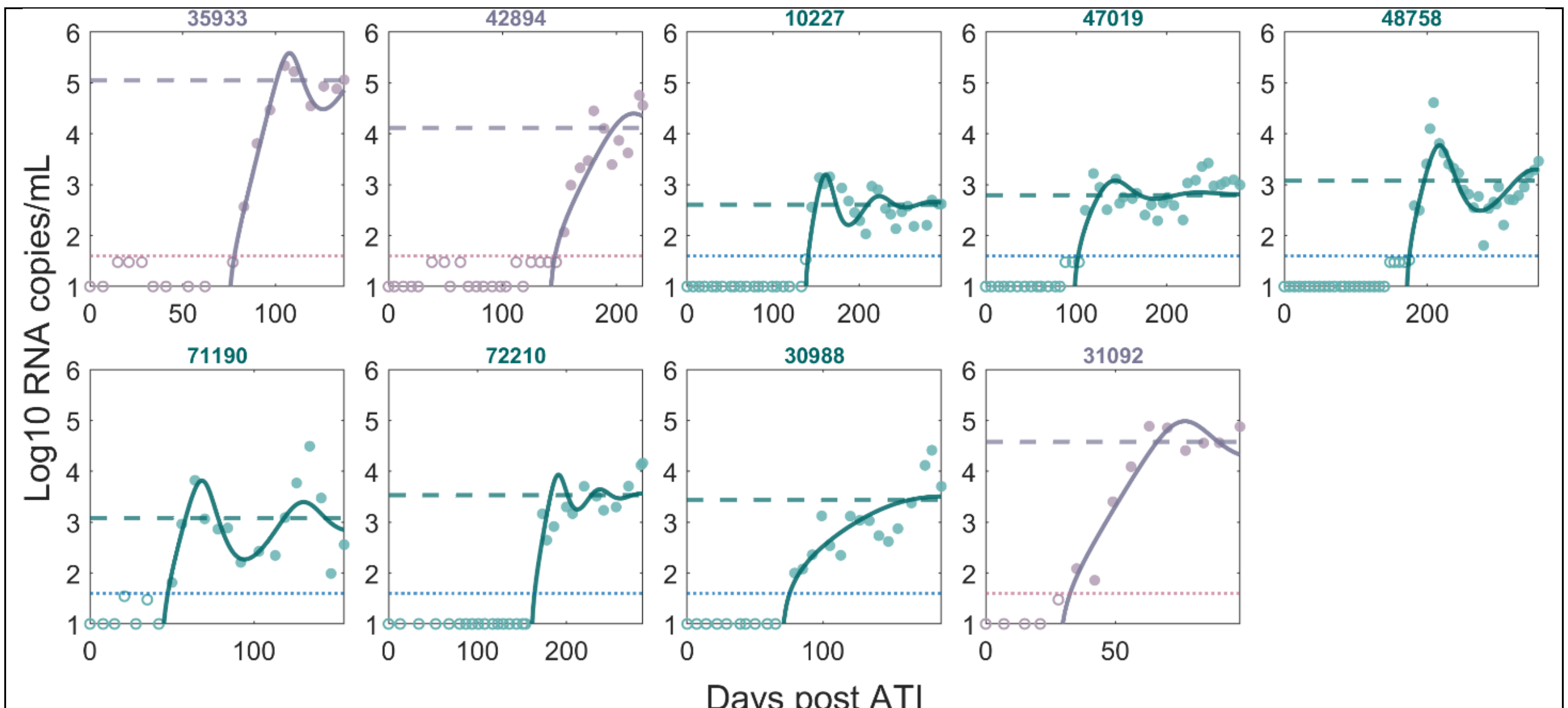

Figure S1. $V_{\text{set point}}$ (given by Equation S1 and shown by a horizontal dashed line) approximates the viral set point well. All viral load trajectories (solid line) either approach or oscillate about $V_{\text{set point}}$.

By averaging over the viral load from the last N-days (N = 7 to 84 days) of observation and using that as an estimate of the viral set point, we show that the viral set point robustly correlates with stemness markers. The correlation with %Ki67+ is strongest using the last week of viral load observations, e.g., the last 2 viral load observations, and the correlation with %TCF-1+ is strongest using the last 2 weeks of observations, e.g., the last 3 viral load observations, (Fig. S2).

bioRxiv preprint doi: https://doi.org/10.64898/2026.08.28.747758; this version posted September 1, 2026. The copyright holder for this preprint (which was not certified by peer review) is the author/funder. This article is a US Government work. It is not subject to copyright under 17 USC 105 and is also made available for use under a CC0 license.

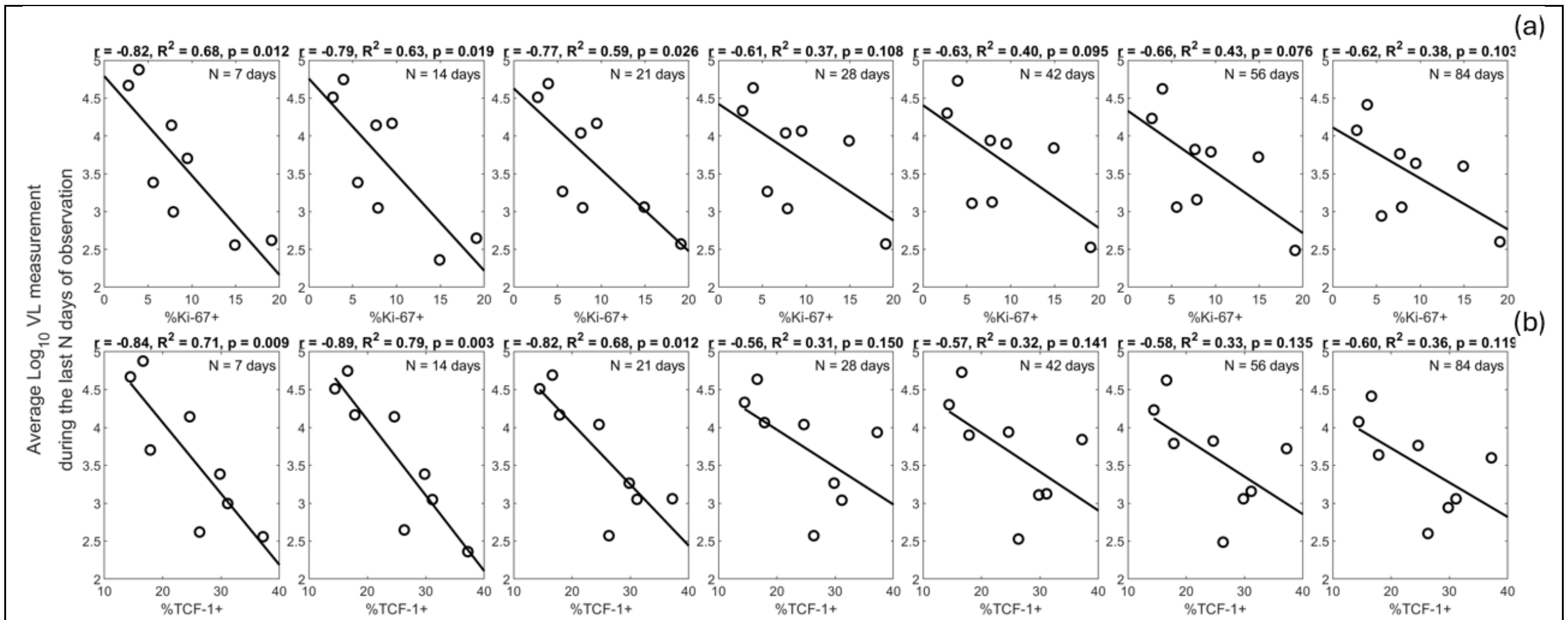

Figure S2. Viral set point correlations with CD8+ T cell markers. Viral set point is estimated using average viral load from the last N days of observation, where N is given at the top right of each panel. (a) Viral set point correlation with %Ki-67+ of non-naïve CD8+ T cells at post-R1. (b) Viral set point correlation with %TCF-1+ of Ki-67+ non-naïve CD8+ T cells at post-R1.
